# Unraveling the Mystery of Fever Source: The Vital Role of Physical Examination in Hospitalized Patients With Emphasis on Peripheral Line-Associated Bloodstream Infections

**DOI:** 10.7759/cureus.42301

**Published:** 2023-07-22

**Authors:** Yoshiro Hadano, Tomohiro Inoue

**Affiliations:** 1 Division of Infection Control and Prevention, Shimane University Hospital, Izumo, JPN; 2 Department of Emergency Medicine, St. Mary's Hospital, Kurume, JPN

**Keywords:** catheter-related bloodstream infection, japan, covid-19, diagnostic error, physical examination

## Abstract

Fever in hospitalized patients requires timely identification of the underlying cause for appropriate treatment. While laboratory tests and imaging studies are valuable, the significance of physical examination should not be overlooked. We present a case of peripheral line-associated bloodstream infection that was readily diagnosed through physical examination during an infectious disease consultation for fever of unknown origin. It is important for busy physicians to prioritize physical examinations to prevent diagnostic errors. Prompt and focused physical examinations contribute to improved patient outcomes and the prevention of healthcare-associated infections.

## Introduction

Prompt identification of fever sources in hospitalized patients is crucial for appropriate management [1]. Physical examination remains essential despite advancements in laboratory and imaging technologies. Busy healthcare settings often limit regular bedside examinations, leading to reliance on laboratory and imaging data. Catheter-related bloodstream infections, including peripheral line-associated bloodstream infections (PLABSIs), are common healthcare-associated infections [2,3]. These infections often arise from the insertion. Any case of undifferentiated fever should be suspected of peripheral line-associated bloodstream infection (PLABSI). However, it is important to note that the incidence of concomitant phlebitis can range from 3% to 63% [4,5]. Moreover, it is important to acknowledge that there are instances where the presence of phlebitis may be overlooked without direct examination. We present a case where the source of fever remained unknown until a focused physical examination was conducted.

## Case presentation

A 65-year-old Japanese woman with no past medical history found in cardiac arrest was admitted to our hospital. In the emergency room, she received adrenaline and achieved return of spontaneous circulation (ROSC) after cardiopulmonary resuscitation (CPR). However, her level of consciousness did not improve, and she remained bedridden. On the day 12th of hospitalization, she had a fever and high C-reactive protein on the 12th day of hospitalization.

On physical examination, her blood pressure was 120/56 mmHg, pulse rate was 98 beats per minute, body temperature was 37.8°C, respiratory rate was 20 breaths per minute, and oxygen saturation was 96% in room air. The physical examination results were unremarkable except for a 2/6 holosystolic murmur in the left fourth intercostal space radiating to the axilla.

Laboratory investigations showed white blood cell count, 8,980/μL (normal range: 3,500-9,000/μL); hemoglobin, 11.3 g/dL (normal range: 13-17 g/dL), and platelet, 159,000/μL (normal range: 140,000-340,000/μL). Evaluation of serum chemistry revealed the following: blood urea nitrogen, 18 mg/dL (normal range: 8.0-20.0 mg/dL); creatinine, 0.6 mg/dL (normal range: 0.6-1.0 mg/dL); sodium, 134 mEq/L (normal range: 0-0.2 mg/dL); potassium, 4.7 mEq/L (normal range: 0-0.2 mg/dL); chloride, 111 mEq/L (normal range: 0-0.2 mg/dL); albumin, 2.5 g/dL (normal range: 4.1-5.1 g/dL); total protein, 6.0 g/dL (normal range: 6.6-8.1 g/dL); aspartate aminotransferase, 45 IU/L (normal range: 13-30 IU/dL); alanine aminotransferase, 40 IU/L (normal range: 10-42 U/L); lactate dehydrogenase, 164 IU/L (normal range: 124-222 U/L); alkaline phosphatase, (normal range: 38-113 mg/dL); total bilirubin, 0.6 mg/dL (normal range: 0.4-1.5 mg/dL); glucose, 111 mg/dL (normal range: 73-109 mg/dL); and C-reactive protein, 8.6 mg/dL (normal range: 0-0.2 mg/dL). Urinalysis was normal. No significant findings were observed on the chest X-ray, and the patient was placed under observation after two sets of blood cultures were obtained.

**Figure 1:**
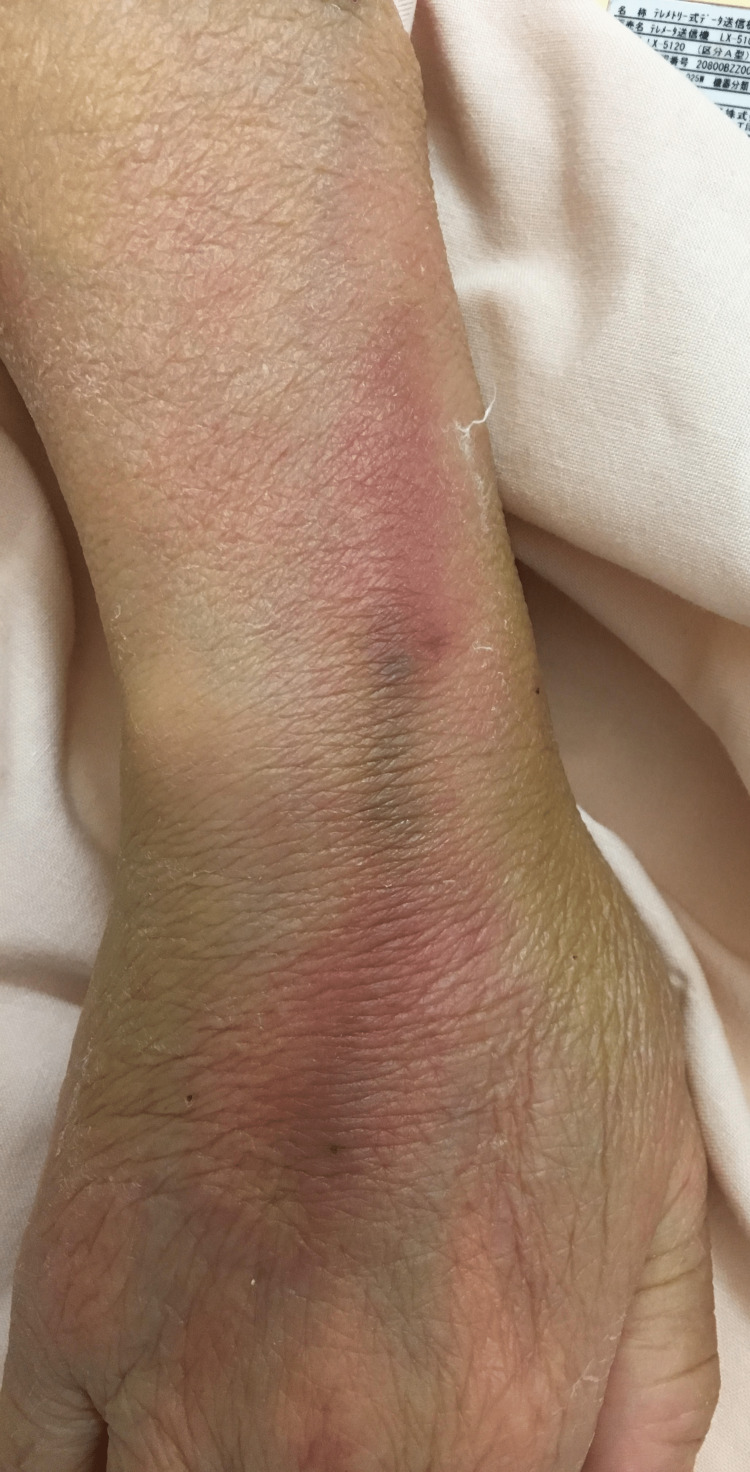
Redness on the left forearm at presentation

On day 13, vancomycin was initiated after two sets of blood cultures were detected. Gram-positive bacillus was detected in two sets of blood cultures. Transthoracic echocardiography showed no vegetation, and computed tomography of the chest and abdomen revealed no lesions that could be the source of infection. Urinary analysis was normal. On day 15, *Bacillus cereus* is identified in two sets of blood cultures, and an infectious diseases consultation was performed due to the unknown focus of* B. cereus* bacteremia. On physical examination, erythema and swelling consistent with the site of the indwelling peripheral catheter and the intravenous route were observed, where the patient was administered daily amino acid preparation (Figure 1).

The patient was diagnosed with peripheral line-associated bloodstream infection (PLABSI) due to *B. cereus*. The isolates of *B. cereus* exhibited susceptibility to meropenem, levofloxacin, minocycline, and vancomycin. However, the isolate from our patient was resistant to ampicillin, cefazoline, ceftriaxone, and cefepime. The peripheral catheter was removed, and a follow-up blood culture was negative. Based on susceptibility results, the patient was successfully treated with a 14-day course of vancomycin (750 mg every 12 hours).

## Discussion

Catheter-related bloodstream infections, including PLABSIs, are frequently encountered as healthcare-associated infections within hospital settings [2,3]. In this case, the patient was receiving amino acid preparations intravenously, and *B. cereus* was detected from two sets of blood cultures, leading to the diagnosis of peripheral line-associated bloodstream infections (PLABSIs). *Bacillus cereus*, which is typically regarded as a contaminant, can also cause peripheral line-associated bloodstream infections [4,5]. In a case-control study utilizing data from eight Japanese hospitals published in 2017, peripheral line insertion (adjusted odds ratio: 213.7, 95% confidence interval: 23.7-1924.6) and amino acid preparations (adjusted odds ratio: 41.6, 95% confidence interval: 4.2-411.7) were identified as risk factors for catheter-related bloodstream infections caused by *B. cereus *[6]*.*

In this case, the physicians were unable to perform daily physical examinations due to their heavy workload. Nevertheless, the infectious diseases physician promptly identified the underlying cause of the fever upon conducting a thorough examination of the patient. For example, in cases of *Staphylococcus aureus* bloodstream infection (SAB), consultations conducted at the bedside have been associated with better outcomes compared to informal telephone consultations [7]. Bedside consultations have been associated with lower mortality rates compared to telephone consultations (odds ratio: 0.09, 95% confidence interval: 0.02-0.49) [7]. Thorough physical examination findings are critical in identifying the underlying cause of fever, including SAB and PLABSIs. These findings highlight the significance of conducting comprehensive physical examinations before diagnosing fever of unknown origin. Based on our experience of a missed case that could have been detected with a physical examination, we strongly advocate for healthcare providers to prioritize and perform a focused physical examination, even in busy settings. It is crucial to actively inspect and evaluate the catheter insertion sites in patients with indwelling catheters to prevent complications.

Fever of unknown origin has multiple potential causes, necessitating detailed medical history taking and physical examination. Previous studies have suggested focusing on conditions such as arthritis, cervical lymphadenopathy, respiratory distress with hypoxia, and ocular symptoms [8]. In cases where physical examination findings are overlooked, it is important to recognize that peripheral line-associated bloodstream infections may be a potential cause of fever of unknown origin. Busy physicians may find it difficult to visit their patients' bedside and perform daily physical examinations. In such cases, decisions may be based on blood sampling data or imaging studies in electronic medical records. While this approach is often successful, in some cases, such as this case, the physical examination remains the key to the diagnosis of the origin of fever. Physicians should be aware that physical examination is still essential and should perform focused and purposeful physical examinations to reduce diagnostic errors [9,10]. Physicians must also be aware of the catheters inserted in their patients daily and remove unnecessary catheters [11]. This is particularly important during COVID-19, when physical examinations may be reduced [12]. Emphasizing the importance of focused and purposeful physical examinations and promoting a multidisciplinary approach can contribute to improved patient outcomes and the prevention of healthcare-associated infections.

## Conclusions

We present a case of PLABSIs with an unknown fever source. This case serves as an example where the failure to perform thorough physical examinations resulted in a diagnosis of fever of unknown origin. The importance of conducting physical examinations promptly becomes evident, as the underlying cause could have been identified with this approach. It highlights the significance of recognizing that physical examination can provide immediate insights and guide diagnosis, emphasizing the need for physicians to prioritize and diligently conduct focused physical examinations to avoid misdiagnosis or delays in identifying the source of fever.
